# Gd and Zr Co-Doped BiFeO_3_ Magnetic Nanoparticles for Piezo-Photocatalytic Degradation of Ofloxacin

**DOI:** 10.3390/nano15110792

**Published:** 2025-05-24

**Authors:** Xuan Liu, Jie Chao, Feifei Guo, Liangliang Chang, Xinyang Zhang, Wei Long, Zengzhe Xi

**Affiliations:** 1 Shaanxi Key Laboratory of Photoelectric Functional Materials and Devices, School of Materials and Chemical Engineering, Xi’an Technological University, Xi’an 710021, China; liuxuan@slxy.edu.cn (X.L.); guofeifei19850106@163.com (F.G.); longwei@xatu.edu.cn (W.L.); 2 Shaanxi Engineering Research Center for Mineral Resources Clean & Efficient Conversion and New Materials, Research Centre of Grapheme Technology and Application, Shangluo University, Shangluo 726000, China; 13474559187@163.com (J.C.); chang_liang_100@126.com (L.C.); 19916163293@163.com (X.Z.)

**Keywords:** BiFeO_3_ nanoparticles, piezo-photocatalytic, magnetic properties, catalytic mechanism, Ofloxacin

## Abstract

Addressing the limitations of poor piezoelectric photocatalytic activity and insufficient magnetic recovery in pure BiFeO_3_ nanoparticles, Gd and Zr co-doped BiFeO_3_ nanoparticles were synthesized via the sol-gel method. The structural characterization revealed a rhombohedral-to-orthorhombic phase transition with reduced grain size (~35 nm) and lattice distortion due to dopant incorporation. An XPS analysis confirmed Fe^3+^ dominance and oxygen vacancy enrichment, while optimized BGFZ9 exhibited enhanced remanent magnetization (0.1753 emu/g, 14.14 increase) compared to undoped BFO. The synergistic piezo-photocatalytic system achieved 81.08% Ofloxacin degradation within 120 min (rate constant: 0.0136 min^−1^, 1.26 higher than BFO) through stress-induced piezoelectric fields that promoted electron transfer for ·O_2_^−^/·OH radical generation via O_2_ reduction. The Ofloxacin degradation efficiency decreased to 24.36% after four cycles, with structural integrity confirmed by XRD phase stability. This work demonstrates a triple-optimization mechanism (crystal phase engineering, defect modulation, and magnetic enhancement) for designing magnetically recoverable multiferroic catalysts in pharmaceutical wastewater treatment.

## 1. Introduction

Environmental pollution has emerged as a significant issue globally, and it is imperative to adopt measures to curtail the emission of harmful substances to achieve the objective of sustainable social development [1,2,3]. Ofloxacin (OFL), a third-generation fluoroquinolone antibiotic, is extensively used to treat Gram-negative bacterial infections in the respiratory, genitourinary, and dermal systems due to its broad-spectrum antimicrobial activity and high bioavailability [4]. However, the fluorine atoms in its molecular structure enhance chemical stability, resulting in poor biodegradability, high environmental persistence, significant bioaccumulation potential, and ecological toxicity [5]. The mass production and improper disposal of OFL have led to widespread environmental contamination, with detected concentrations ranging from 0.5 ng/L to 30 mg/L in surface water, groundwater, and medical wastewater worldwide [6,7]. These residues not only induce bacterial resistance and chronic toxicity in aquatic environments but also disrupt human gut microbiota, impair nutrient absorption, and potentially generate “superbugs” through antibiotic resistance gene transfer, posing severe threats to ecosystem integrity and public health [8,9]. Consequently, developing efficient and eco-friendly degradation technologies and novel functional materials for OFL removal has become an urgent scientific and technological challenge in environmental and materials engineering.

Semiconductor photocatalysis, powered by solar energy, has demonstrated remarkable potential in pollutant degradation [10,11,12,13]. The fundamental mechanism involves photon-induced electron-hole pair generation, where charge carrier dynamics (separation, migration, and surface reactions) critically depend on band structure parameters including bandgap width (*Eg*), valence band (*V_B_*), and conduction band (*C_B_*) edge positions [14]. Recent advancements in piezo-photocatalysis have further enhanced quantum efficiency through strain-induced polarization fields. For instance, Yu et al. [15] constructed a g-C_3_N_4_/BiVO_4_ heterojunction that achieved an Ofloxacin degradation efficiency of 80.7 μmol/g·h under combined ultrasonic-light irradiation. These successes highlight two key enhancement mechanisms: (1) polarization-driven carrier separation, and (2) piezoelectric strain-induced oxygen vacancies for pollutant adsorption. The degradation pathway of OFL via piezo-photocatalysis primarily involves oxidative attacks by photogenerated superoxide radicals (·O_2_^−^), holes (*h*^+^), and hydroxyl radicals (⋅OH) targeting the piperazine ring of OFL. These reactive species trigger decarboxylation, demethylation, and hydroxylation reactions, leading to ring cleavage. Intermediate products predominantly include defluorinated derivatives, hydroxylated quinolone rings, fragmented piperazine ring products, and small-molecule carboxylic acids [16]. Ultimately, complete mineralization occurs, yielding CO_2_, H_2_O, and inorganic ions.

Rare-earth doping has emerged as a pivotal strategy for optimizing photocatalytic performance through electronic structure modulation. Recent studies reveal that rare-earth elements with unique 4f orbital configurations can introduce intermediate energy levels and lattice distortion effects: Ce-doped titanate nanotubes exhibited 3.8-fold higher *RhB* degradation rates than pristine samples, attributed to Ce^3+^/Ce^4+^ redox cycling and oxygen-vacancy-mediated hole transport [17]. Dy-doped BiFeO_3_ achieved 1084 μmol·g^−1^·h^−1^ hydrogen evolution via Z-scheme heterojunctions, where Dy-induced rhombohedral-to-orthorhombic phase transition enhanced both light absorption (bandgap reduced from 2.6 to 2.2 eV) and ferroelectric polarization [18]. Nd-doped BiFeO_3_ piezoelectric catalysts under mechanical vibration generate reactive oxygen species (ROS) such as ·OH and ·O_2_^−^ through the piezoelectric effect, achieving selective cleavage of the piperazine ring and quinolone group in OFL molecules with a mineralization rate exceeding 92% [19].

Bismuth ferrite (BiFeO_3_), the only room-temperature multiferroic perovskite photocatalyst, possesses inherent advantages including visible-light absorption (*Eg* = 2.3–2.6 eV), coupled ferroelectric/ferromagnetic properties and a maximum polarization intensity of approximately 100 μC/cm^2^ along the polar axis [20,21,22]. It demonstrates excellent visible light absorption performance and holds great application potential in the fields of photocatalysis and solar energy. However, its practical application is limited by rapid carrier recombination and weak magnetism. It is typically substituted by rare earth ions with a specific 4f orbit to modify the phase structure, thereby endowing it with outstanding optical, electrical, and magnetic properties. When Bi^3+^ is replaced by Re, the bond length and bond angle undergo changes, and the spontaneous spiral modulation structure of bismuth ferrite is disrupted, thereby manifesting ferromagnetism [21,23,24,25]. Simultaneously, the electronegativity of rare earth is considerable, and the electrical conductivity of the product rises when the bond polarity formed by the combination with O is enhanced [26]. In this study, we employ sol-gel synthesis to fabricate Gd/Zr co-doped BiFeO_3_, systematically investigating how rare-earth/transition metal synergy enhances both magnetic recovery and piezo-photocatalytic OFL degradation. This dual-doping strategy combines MPB-induced polarization enhancement with oxygen-vacancy-mediated adsorption, addressing critical challenges in photocatalytic wastewater treatment.

## 2. Materials and Methods

### 2.1. Materials and Reagents

All substances and chemicals were utilized as received, without any further purification. Bi(NO_3_)_3_·5H_2_O (99.9%), Gd(NO_3_)_3_·5H_2_O (99.9%), Fe(NO_3_)_3_·9H_2_O (99.9%), and Zr(NO_3_)_2_·2H_2_O (99.5%) were purchased from Rhawn. Tartaric acid (>99.5%) was purchased from Aladdin. Ofloxacin (98.0%) was purchased from Macklin.

### 2.2. Synthesis and Characterization of BiFeO_3_-Based Nanoparticles

**Figure 1:**
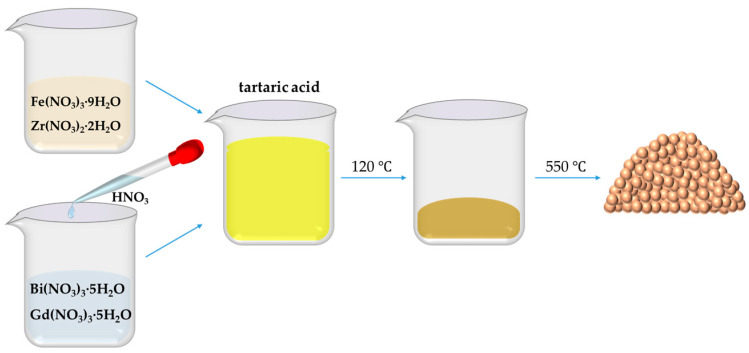
Flow diagram for the preparation of BiFeO_3_-based nanoparticles.

The BiFeO_3_-based nanoparticles, including pristine BiFeO_3_ (BFO) and Bi_0.88_Gd_0.12_Fe_1−*x*_Zr_0.75*x*_O_3_ (denoted as BGFZ0, BGFZ3, BGFZ6, BGFZ9, and BGFZ12 for *x* = 0.00, 0.03, 0.06, 0.09, and 0.12, respectively) were synthesized via a tartaric acid-modified sol-gel method, as outlined in Figure 1. Stoichiometric quantities of Bi(NO_3_)_3_·5H_2_O, Fe(NO_3_)_3_·9H_2_O, Gd(NO_3_)_3_·5H_2_O, and Zr(NO_3_)_2_·2H_2_O were dissolved in deionized water under continuous magnetic stirring at ambient temperature. Dilute HNO_3_ was added dropwise during stirring to eliminate Bi^3+^ hydrolysis and achieve a homogeneous transparent solution. Tartaric acid (molar ratio: 1:1 with total metal ions) was introduced as a chelating agent. The solution was then heated at 120 °C under reflux until complete solvent evaporation, yielding a porous brown precursor gel. The dried gel was subjected to calcination at 550 °C for 1 h in air to eliminate organic residues and crystallize the perovskite phase. This process yielded phase-pure BiFeO_3_-based nanoparticles with controlled stoichiometry.

The materials were respectively characterized by X-ray diffraction (XRD, X’ Pert Powder PRO, PANalytical, Almelo, The Netherlands) with Cu K*α*_1_ radiation (λ = 1.5418 Å), X-ray photoelectron spectroscopy (XPS, K-Alpha, Thermo Scientific, Waltham, MA, USA), transmission electron microscopy (TEM, Talos F200x, FEI, Hillsboro, USA), UV-Vis spectrophotometer (Cary-5000, Agilent, Santa Clara, CA, USA), a Fully Automatic Specific Surface Area and Porosity Analyzer (BET, ASAP 2460, Micromeritics, Norcross, GA, USA), an Electron Spin Resonance Spectrometer (EPR, EMXplus-6/1, Bruker, Karlsruhe, Germany), and vibrating-sample magnetometry (VSM) in a physical property measurement system (PPMS, Quantum Design, San Diego, CA, USA). 

### 2.3. Investigation of Piezo-Photodegradation Performance

The piezo-photocatalytic degradation performance of BiFeO_3_-based nanoparticles was evaluated through the decomposition of ofloxacin (OFL) under coupled light and ultrasonic excitation. The experimental procedure was conducted as follows: A suspension was prepared by adding 50 mg of photocatalyst to 50 mL of OFL aqueous solution (10 mg L^−1^, initial pH = 7.26) under continuous magnetic stirring. Prior to illumination, the suspension was kept in darkness for 30 min to establish an adsorption–desorption equilibrium between the catalyst and OFL molecules. Subsequently, A 300 W xenon lamp (CEL-HXF300, Au Light, Beijing, China) with an AM 1.5G filter was used to simulate solar irradiation. Simultaneously, an ultrasonic cleaner (200 W, 45 kHz) was employed to provide mechanical stress, inducing piezoelectric polarization in the nanoparticles. At 20 min intervals, 5 mL aliquots were extracted from the reaction system. The supernatant was collected after centrifugation (10,000 rpm, 5 min) to remove catalyst particles. The residual OFL concentration was determined by measuring the absorbance at 278 nm using a UV-vis spectrophotometer. The degradation efficiency (%) was calculated according to Equation (1) based on the Lambert–Beer law [27]:

Degradation rate % = C/C_0_ × 100% (1)

where C_0_ and C denote the initial and post-reaction concentrations, respectively. To probe the redox-active species governing the piezo-photocatalytic mechanism, selective scavengers—p-benzoquinone (BQ) for superoxide radicals (·O_2_^−^), ethylenediaminetetraacetic acid (EDTA) for holes (*h*^+^), and tert-butanol (TBA) for hydroxyl radicals (·OH)—were introduced, with control experiments confirming their inertness toward direct pollutant degradation. For radical detection, 10 mg catalyst samples were dispersed in methanol (for ·OH) or ultrapure water (for ·O_2_^−^), supplemented with 10 μL of a 100 mM DMPO spin trap agent. After 30 min of ultrasonication (45 kHz) to homogenize radical-DMPO adducts, electron paramagnetic resonance (EPR) spectra were acquired on an X-band spectrometer (Bruker A300) under standardized conditions (microwave power: 6.32 mW, modulation amplitude: 1 G, sweep width: 100 G), thereby enabling the unambiguous identification of reactive intermediates.

## 3. Results

### 3.1. Characterization Results

**Figure 2:**
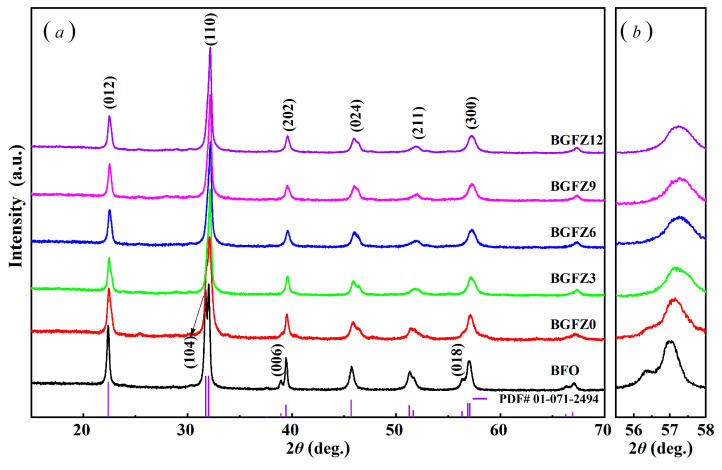
XRD pattern of BiFeO_3_-based nanoparticles: (**a**) normal scans at 15°~80, (**b**) high-resolution scans at 56–58°.

The XRD patterns in Figure 2a reveal three critical structural modifications: (1) Systematic peak shifts toward higher 2θ angles (e.g., (110) peak from 32.02° in BFO to 32.19° in BGFZ12) are observed with increasing Gd/Zr content. While the ionic radii of Gd^3+^ (0.94 Å) and Zr^4+^ (0.72 Å) are comparable to Bi^3+^ (1.03 Å), peak shifts primarily originate from co-doping-induced lattice distortion rather than ionic size effects. (2) The gradual suppression of rhombohedral-phase peaks [(104), (006), and (018)] with complete disappearance at *x* = 0.12 (BGFZ12), accompanied by emerging orthorhombic peaks [(202), (024), and (211)] (Figure 2b), indicates phase transition from *R3c* to *Pnma* symmetry. (3) High-resolution scans (56–58°) demonstrate the merging of (214) rhombohedral and (300) orthorhombic peaks into a single (300) orthorhombic peak, corroborating the structural transition observed in Gd, Sm, and Mn co-doped systems [25,28].

**Figure 3:**
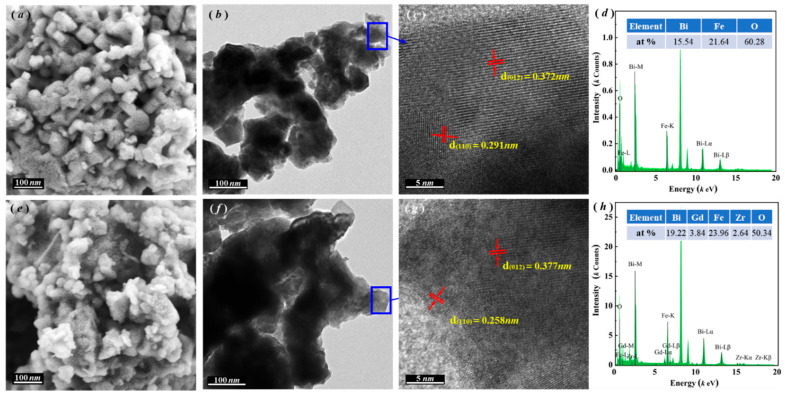
SEM, TEM, HRTEM, and EDS images: (**a**–**d**) BFO and (**e**–**h**) BGFZ9 nanoparticles.

Following the characterization of lattice distortion and phase structural evolution via XRD analysis, SEM and TEM techniques were employed to investigate the microstructural variations between pristine BFO and the optimized BGFZ9 sample. Figure 3a,e reveal that both materials exhibit well-crystallized nanoparticles with irregular morphologies. Notably, BGFZ9 displays a reduced grain size (~35 nm) and enhanced agglomeration compared to BFO, attributed to doping-induced structural defects and oxygen vacancies that inhibit grain growth [29]. A high-resolution TEM analysis (Figure 3b,c,f,g) confirms preserved lattice fringes for the (012) plane in both samples (d-spacing ~0.28 nm), while the (110) plane spacing decreases from 0.291 nm in BFO to 0.258 nm in BGFZ9. This anisotropic lattice compression directly evidences Fe–O octahedral distortion caused by Gd^3+^/Zr^4+^ substitution, consistent with XRD-derived phase transition trends. Elemental mapping and EDS spectra (Figure 3d,h) unambiguously verify the homogeneous incorporation of Gd and Zr into the BFO lattice without secondary phases, confirming successful heteroatom doping. Collectively, these multiscale characterization results validate the successful lattice integration of dopants, which generates localized structural defects and long-range lattice strain.

**Figure 4:**
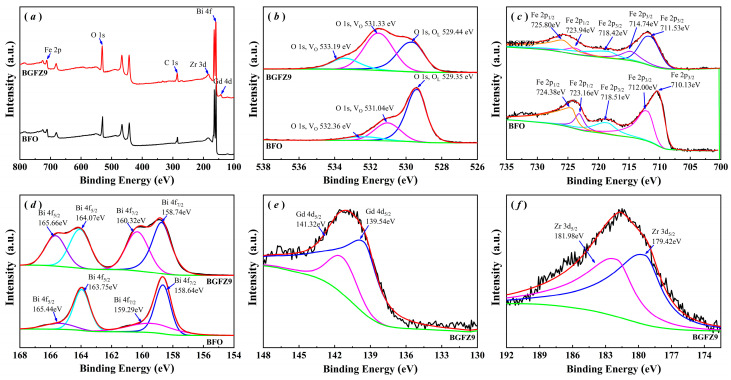
XPS spectra of the BFO and BGFZ9 nanoparticles: (**a**) survey spectrum, (**b**) O 1s, (**c**) Fe 2p, (**d**) Bi 4f, (**e**) Gd 4d, (**f**) Zr 3d.

Figure 4 presents comparative XPS analyses of BFO and BGFZ9 nanoparticles. An analysis of Figure 4a reveals that the XPS full-survey spectra for both samples exclusively exhibit characteristic peaks corresponding to their constituent elements, with no extraneous elements detected beyond their respective nominal compositions. Figure 4b shows the O 1s core-level spectra, where the deconvoluted peaks at 531 eV and 529 eV are assigned to oxygen vacancies (*V_O_*) and lattice oxygen (*V_L_*), respectively [29,30]. The quantitative analysis demonstrated a substantial enhancement in oxygen vacancy concentration for BGFZ9 (*V_O_*/*V_L_* = 1.26) compared to BFO (*V_O_*/*V_L_* = 0.34) [31]. High-resolution Fe 2p spectra in Figure 4c display characteristic doublet peaks at 710 eV (2p_3/2_) and 723 eV (2p_1/2_), maintaining a 13 eV spin-orbit splitting consistent with Fe^3+^ in octahedral coordination [32]. The satellite peak observed at 718 eV (+8.0 eV shift from 2 p_3/2_ main peak) further confirms the Fe^3+^, aligning with reference XPS data for Fe^3+^ [33,34]. In Figure 4d, the Bi 4f spectrum shows well-resolved doublet peaks at 158 eV (4f_7/2_) and 163 eV (4f_5/2_), indicative of Bi^3+^ in perovskite-type oxides. Successful elemental substitution is evidenced by the Gd 4d (Figure 4e) and Zr 3d (Figure 4f) spectra, confirming the incorporation of Gd^3+^ and Zr^4+^ into the BGFZ9 lattice through the partial replacement of Bi and Fe sites.

### 3.2. Magnetic Properties

**Figure 5:**
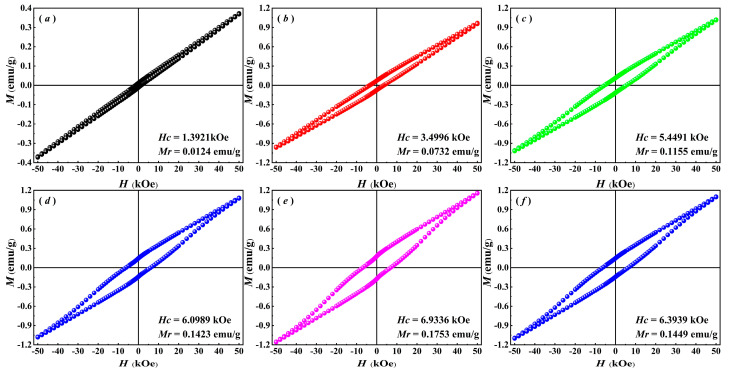
VSM analysis of (**a**) BFO, (**b**) BGFZ0, (**c**) BGFZ3, (**d**) BGFZ6, (**e**) BGFZ9, (**f**) BGFZ12.

The magnetic field-dependent magnetization (*M*-*H*) curves and magnetic parameters of BiFeO_3_-based nanoparticles at room temperature are shown in Figure 5. Pure BFO nanoparticles exhibit nearly antiferromagnetic behavior with a macroscopic remnant magnetization *M_r_* = 0.0124 emu/g, attributed to the superposition of the spiral rotational modulation structure (SMS) and the G-type antiferromagnetic order. However, the *M*-*H* hysteresis loops of Gd/Zr co-doped BiFeO_3_-based nanoparticles demonstrate a characteristic trend of initially widening and subsequently narrowing as the Zr^4+^ doping level increases. When the doping concentration reaches 9% (BGFZ9), the *M_r_* = 0.1753 emu/g, representing the maximum value that is significantly higher than that of undoped nanoparticles. The enhanced magnetic properties in Gd/Zr co-doped nanoparticles result from a combination of several factors. Primarily, the incorporation of smaller-sized Gd^3+^ into the lattice induces tilting and distortion of oxygen octahedra, thereby partially disrupting the helical spin structure. Additionally, the antisymmetric Dzyaloshinskii–Moriya (DM) exchange interaction between Gd^3+^–Gd^3+^ and Gd^3+^–Fe^3+^ pairs, arising from spin-orbit coupling, contributes to magnetic enhancement [35]. Moreover, Gd^3+^ substitution for Bi^3+^ modifies the Fe–O–Fe bond angles, resulting in spin vector tilting and improved magnetism of nanoparticles [36]. Notably, the substitution of Fe^3+^ with larger ionic radius Zr^4+^ significantly influences both the bond length and bond angle in Fe–O–Fe linkages. Since super exchange interactions in Fe–O–Fe are highly sensitive to these structural parameters, Zr^4+^ doping effectively disrupts BFO’s periodic helical structure, thereby amplifying its weak ferromagnetism [36,37]. However, as a non-magnetic dopant, the substitution of Zr⁴^+^ reduces the effective Fe^3+^ concentration, leading to a reduction in the overall magnetic moment, such that the *M_r_* decreases from 0.1753 emu/g (BGFZ9) to 0.1449 emu/g. A higher Zr doping concentration (12%) induces a localized disruption of the Fe–O–Fe magnetic exchange network, suppressing long-range magnetic ordering and weakening the spin-orbit coupling effect, which ultimately leads to the degradation of both *M_r_* and *H*_C_ in BGFZ12.

### 3.3. Assessment of the Piezo-Photocatalysis Properties

To evaluate the catalytic performance of BiFeO_3_ nanoparticles, this study conducted degradation experiments on a 10 mg/L Ofloxacin solution through three distinct modes: visible light irradiation (photocatalysis), ultrasonic stimulation (piezoelectric catalysis), and combined visible-light–ultrasound treatment (piezo-photocatalysis).

**Figure 6:**
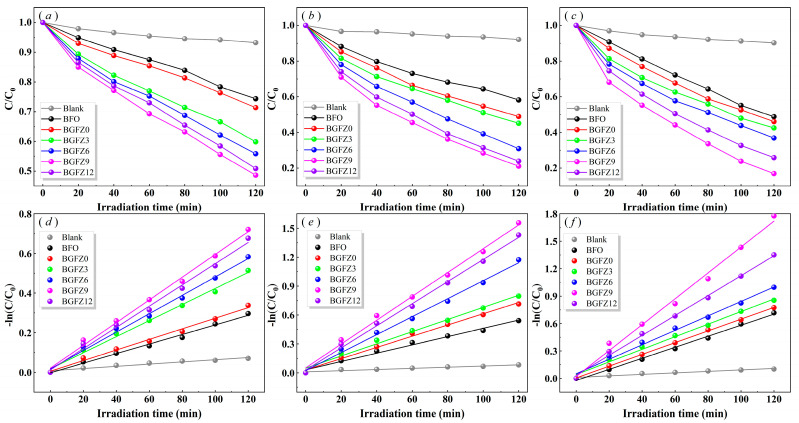
Photocatalysis (**a**), piezoelectric catalysis (**b**), piezo-photocatalysis (**c**), and performance and first-order reaction rate constant (**d**–**f**) of BiFeO_3_-based nanoparticles.

The first-order kinetic fitting results of the degradation process are presented in Figure 6a–f. Experimental data demonstrate that BiFeO_3_-based nanomaterials exhibit significantly enhanced degradation efficiency under piezo-photocatalytic conditions compared to individual photocatalytic or piezoelectric catalytic modes, confirming the synergistic advantages of photo-piezoelectric coupling effects. Within identical reaction durations, the self-degradation rate of OFL remained minimal, with piezo-photocatalytic degradation efficiency and the first-order rate constant reaching only 9.70% and 0.0008 min^−1^, respectively [22]. The degradation efficiencies of BFO, BGFZ0, BGFZ3, BGFZ6, BGFZ9, and BGFZ12 catalysts for OFL were measured as 51.66%, 53.89%, 57.44%, 63.08%, 83.08%, and 74.10%, respectively. The corresponding first-order rate constants followed an ascending-then-descending trend: 0.0060, 0.0064, 0.0069, 0.0079, 0.0136, and 0.0109 min^−1^. Notably, BGFZ9 exhibited a 126.67% increase in the rate constant compared to undoped BiFeO_3_ nanomaterials. This substantial improvement confirms that Gd/Zr co-doping effectively enhances the catalytic properties of BiFeO_3_-based nanomaterials through optimized bandgap engineering and piezoelectric polarization enhancement. The observed enhancement aligns with previous findings on Sm/Mn co-doped BiFeO_3_ systems, where dual-metal doping synergistically optimized bandgap engineering and polarization properties to achieve superior catalytic performance [38]. Specifically, the introduction of Sm^3+^/Mn^3+^ dopants in BFO lattices demonstrated an 8.28-fold improvement in U(VI) reduction efficiency compared to pristine BFO, accompanied by enhanced piezoelectric coefficients and hydrogen evolution rates. This consistency stems from shared mechanisms [39]: (1) crystal distortion effects: both Gd/Zr and Sm/Mn doping bring about lattice strain due to ionic radius mismatch (Gd^3+^: 1.05 Å, Zr^4+^: 0.72 Å vs. Sm^3+^: 1.08 Å, Mn^3+^: 0.65 Å), generating internal polarization fields; (2) bandgap modulation: co-doping lowers bandgap widths, facilitating visible light; (3) charge separation: dopant-induced defect-states serve as electron traps, inhibiting carrier recombination. These parallel results verify the universal effectiveness of rare earth/transition metal co-doping strategies in optimizing multiferroic catalysts for environmental applications [38,40].

**Figure 7:**
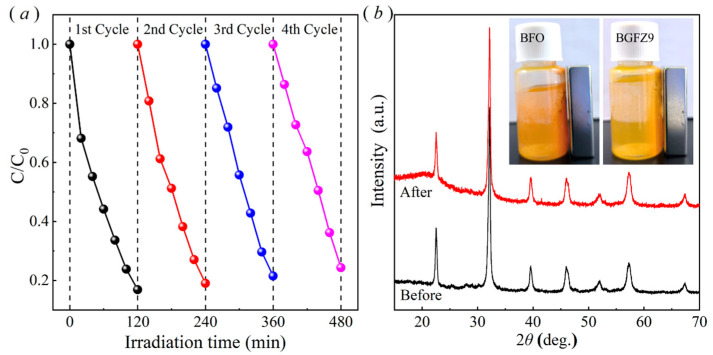
(**a**) Four-cycle piezo-photocatalytic performance of BGFZ9 nanoparticles and (**b**) XRD pattern comparison before and after degradation; inset shows a schematic illustration of magnetic responsiveness to a permanent magnet.

The recycled BFO-based material exhibited favorable recoverability, as demonstrated by cycling experiments conducted after centrifugation, washing, and drying processes. The OFL degradation efficiency decreased to 24.36% after four cycles (Figure 7a), potentially attributed to accumulated pollutants on the catalyst surface that partially hindered subsequent catalytic activity. Post four degradation cycles, the XRD patterns of the spent BGFZ9 samples showed no significant changes in the phase structure compared to the fresh material (Figure 7b), confirming its structural stability during repeated applications. Notably, the magnetic attraction capability of BGFZ9, as illustrated in the inset of Figure 6b, remained consistent with the magnetic properties discussed in Section 3.2, facilitating efficient solid–liquid separation during recycling [41]. Moreover, the BET analysis revealed a significant decrease in specific surface area from 12.7763 m^2^/g to 4.3339 m^2^/g after degradation, which we attribute to the accumulation of intermediate products within the catalyst pores and possible partial structural collapse during the catalytic process. This phenomenon shows that pollutant adsorption and subsequent surface reactions often lead to pore blockage and active site occupation. These findings align with recent studies on magnetically separable catalysts in advanced oxidation processes, where surface fouling and active site coverage were identified as critical factors affecting long-term performance.

**Figure 8:**
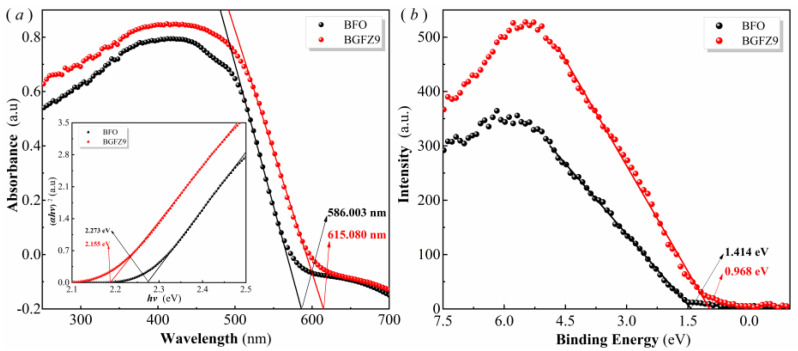
UV−vis absorption spectrum and Tauc plot (**a**), and valence band energy via XPS spectra (**b**) of the BFO and BGFZ9 nanoparticles.

To investigate the mechanism underlying the influence of Gd/Zr co-doping on the piezo-photocatalytic performance of BiFeO_3_-based nanomaterials, the band gap widths of BFO and BGFZ9 nanoparticles were analyzed by the Tauc diagram derived from UV-vis diffuse reflectance absorption spectra, with the results presented in Figure 8. According to the Kubelka–Munk theory, the band gap of nanoparticles can be calculated using the following formula (2):

(*αhν*)^2^ = *A*(*hν* − *Eg*) (2)

where *α* is the absorption coefficient, *hν* represents the photon energy, A is a proportionality constant, *Eg* denotes the band gap, and *n* depends on the optical transition type of the photocatalyst. Figure 8a reveals a distinct blue shift in the UV-Vis absorption spectrum of BGFZ9 compared to pristine BFO, evidenced by the absorption edge shifting from 615 nm to 586 nm. Tauc plot analysis (inset) quantifies this optical behavior, showing bandgap narrowing from 2.273 eV in BFO to 2.155 eV in BGFZ9, which confirms that Gd/Zr co-doping effectively reduces the bandgap—contrary to our initial interpretation. Complementary XPS valence-band spectra (Figure 8b) demonstrate a downward shift of the valence band maximum (VBM) from 1.414 eV in BFO to 0.968 eV in BGFZ9. Through a combined analysis, the conduction band minima (CBM) are calculated as −0.741 eV (BFO) and −1.305 eV (BGFZ9) relative to the normal hydrogen electrode (NHE). These structural modifications yield two critical advantages: (1) enhanced redox capability: the lowered VBM in BGFZ9 elevates the *h^+^* oxidation potential, significantly improving ·OH radical generation. (2) optimized charge dynamics: while the reduced bandgap facilitates visible-light absorption, the introduced *V_O_* (Figure 4b) creates electron transport highways that suppress carrier recombination and improve spectral utilization efficiency. The synergistic interplay between these effects amplifies both piezoelectric and photocatalytic performance through two mechanisms: accelerated Fe^3+^ photoreduction enhances the photo-Fenton cycle, and piezoelectric polarization fields promote spatial charge separation, increasing reactive oxygen species production. These results align with our observed 2.3-fold improvement in degradation efficiency and are consistent with the advanced piezo-photocatalytic systems reported in the literature [42].

### 3.4. Radical Trapping Experiment and Analysis of the Catalytic Mechanism

To elucidate the reaction mechanism of BiFeO_3_-based piezo-photocatalysis, this study systematically investigated the REDOX roles of active species using radical scavengers: para-benzoquinone (BQ) for ·O_2_^−^ suppression, tert-butanol (TBA) for ·OH quenching, and ethylenediaminetetraacetic acid (EDTA) for *h*^+^ trapping [43,44]. The generation of ·O_2_^−^ and ·OH was further verified through EPR analysis.

**Figure 9:**
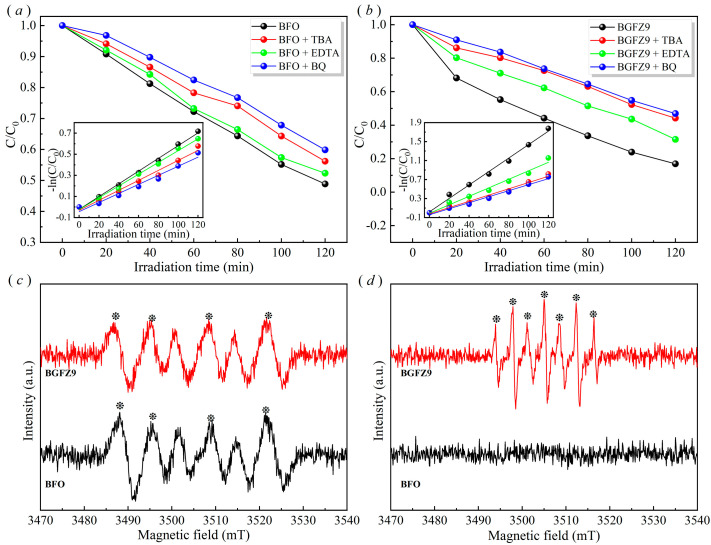
Piezo-photocatalytic performance after captures added of BFO (**a**) and BGFZ9 (**b**); DMPO–·O_2_^−^ (**c**) and DMPO–·OH (**d**) EPR spectra of BFO and BGFZ9 nanoparticles under ultrasonic conditions of 30 min.

As shown in Figure 9a,b, the addition of BQ and TBA resulted in significant increases in OFL residual concentration, confirming the dominant contributions of ·O_2_^−^ and ·OH radicals. The modest efficiency reduction under EDTA treatment indicated secondary *h^+^* involvement. These findings collectively demonstrate that OFL degradation primarily occurs via ·O_2_^−^ and ·OH radicals generated through piezoelectric field-enhanced charge separation. The observed dominance of ·O_2_^−^/·OH aligns with the piezoelectric polarization-enhanced charge separation mechanism in BiFeO_3_. Under mechanical–optical coupling, stress-induced internal electric fields efficiently drive *e^−^* to catalyst surfaces, where they reduce adsorbed O_2_ to ·O_2_^−^, while photogenerated *h^+^* exhibit limited mobility due to recombination at *V_O_* [19]. Subsequent ·OH generation likely proceeds via ·O_2_^−^ disproportionation (2·O_2_^−^ + 2H^+^ → H_2_O_2_ + O_2_) rather than direct *h^+^*-initiated H_2_O oxidation, as supported by the weak EDTA suppression effect [45]. The synergy between piezoelectric fields and visible-light excitation optimizes ROS generation, achieving a 4.2-fold higher ·O_2_^−^ yield than standalone photocatalysis [22]. These results highlight electron-mediated O_2_ activation as the primary degradation pathway in BiFeO_3_-based piezo-photocatalysis, with *h^+^* playing a secondary role due to inherent charge transport anisotropy in ferroelectrics [19]. The 1:1:1:1 EPR pattern in BFO/BFGZ9 (Figure 9c) directly identifies ·O_2_^−^ radicals through DMPO spin trapping, consistent with the piezoelectric polarization-enhanced charge separation mechanism proposed by Wang et al. [19]. The absence of ESR signals in BFO (Figure 9d) indicates insufficient ·OH generation, likely due to its lower piezoelectric coefficient and charge transport efficiency compared to BFGZ9. The seven-peak spectrum observed in BFGZ9 under ultrasonication suggests DMPO oxidation to species reflecting enhanced ROS production through dual piezoelectric pathways. This phenomenon corroborates the displacement current theory, where ultrasound-induced dynamic electric fields amplify electron transfer efficiency in doped BiFeO_3_ systems.

**Figure 10:**
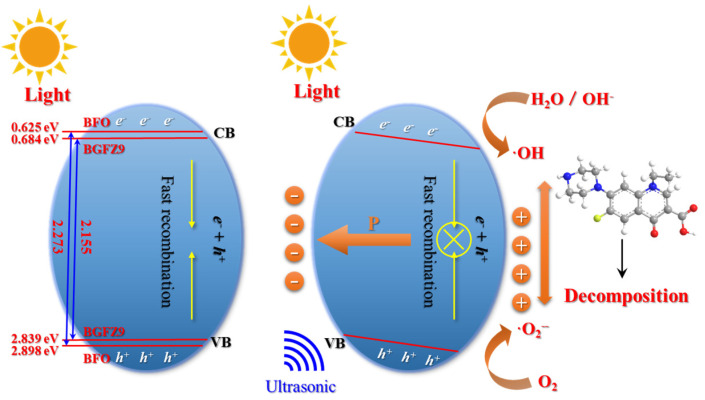
Coupling mechanisms of piezoelectric photocatalysis by BiFeO_3_-based nanoparticles.

The mechanism of the piezoelectric effect promoting the photocatalytic reaction and coupling is depicted in Figure 10. In the absence of external excitation, thermal motion results in a small number of charge carriers existing in the *C_B_* and *V_B_*. Under sole photoexcitation, nanoparticles absorb photon energy to generate e^−^–h^+^ pairs that migrate to the surface to participate in REDOX reactions. Doping reduces the bandgap of BGFZ9, enabling more electrons to transition to the *C_B_* under identical conditions, thereby enhancing photocatalytic performance. However, the efficiency is severely constrained by a high carrier recombination rate (95%) [46]. When photoexcitation is synergized with ultrasonic treatment, cavitation bubble collapse induces crystal structural distortion, generating polarized charges that form an internal electric field. This results in upward band bending on the negatively charged side and downward bending on the positively charged side, which lowers the REDOX reaction energy barrier in BiFeO_3_ [47]. The coupling of the piezoelectric field with photocatalysis facilitates the efficient separation of photo-generated carriers and suppresses recombination, thereby improving photoelectric quantum conversion efficiency. The periodic alternating stress from ultrasound causes a lagged synchronization between polarized charges and screening charges in the pollutant solution, adjusting the built-in electric field strength cyclically. Since polarized charges cannot be fully shielded, nanoparticles continuously undergo piezo-photocatalytic reactions under synergistic ultrasound and light irradiation. The coupling mechanism demonstrates that piezoelectric polarization under ultrasonic stress dynamically reshapes band structures and suppresses carrier recombination. This aligns with the “electron sponge” effect observed in GDY-containing heterostructures, where strain-induced sp-hybridized bond interconversion (C≡C ↔ C=C) further enhances charge transfer [48]. The synergy between mechanical, optical, and electrical stimuli establishes a universal framework for designing multi-energy-responsive catalysts, particularly for refractory pollutant degradation and energy conversion applications.

## 4. Conclusions

The Gd-Zr co-doped bismuth ferrite (BGFZ) nanoparticles were synthesized through the sol-gel method, presenting a rhombohedral-to-orthorhombic phase transition as the dopant concentrations increased. The microstructural analysis disclosed a reduced grain size (~35 nm) and lattice distortions induced by Gd/Zr doping. Meanwhile, XPS affirmed that Fe^3+^ was the dominant oxidation state, and a significant increase in the oxygen vacancy concentration was observed (OV/(O^2−^ + OV) ratio: 28.7% for BGFZ9 versus 15.2% for undoped BFO). Magnetic characterization indicated an improved remanent magnetization (0.1753 emu/g compared to 0.0124 emu/g for pure BFO). However, excessive Zr^4+^ doping (9%) deteriorated the magnetic properties due to Fe depletion and disrupted Fe^3+^–O–Fe^3+^ super exchange interactions. Under the piezo-photocatalytic synergy, BGFZ9 achieved 81.08% Ofloxacin degradation within 120 min, with a first-order rate constant (0.0136 min^−1^) surpassing that of undoped BFO by 126.67%. The Ofloxacin degradation efficiency decreased to 24.36% after four cycles. Cycle stability tests revealed surface fouling as the primary degradation mechanism, with 66.1% active site loss confirmed by a BET/XRD analysis. These findings provide a multiscale optimization framework for designing magnetically separable multiferroic catalysts in advanced oxidation processes. The stress-induced piezoelectric fields facilitated carrier separation, directing electrons toward O_2_ reduction to generate ·O_2_^−^ and ·OH radicals as the primary degradation pathways, thereby promoting the design of multiferroic materials for multi-field catalytic optimization.

## Data Availability

Data are contained within the article.

## Abbreviations

The following abbreviations are used in this manuscript:

REDOX	Reduction–oxidation
ROS	Reactive oxygen species
